# Heterogeneity in dual-career stress: an integrative person-centered and distribution-sensitive analysis of its asymmetric effects on adolescent football players

**DOI:** 10.3389/fpsyg.2026.1789877

**Published:** 2026-03-06

**Authors:** Zhengri Quan, Guannan Liu, Hang Yin, Dan Pang

**Affiliations:** 1 School of Physical Education and Health, Changchun Normal University, Changchun, China; 2 School of Sports Medicine, Anshan Normal University, Anshan, China; 3 School of Sports Science, China Three George University, Yichang, China

**Keywords:** academic performance, adolescent athletes, dual career, latent profile analysis, quantile regression, sports burnout, stress heterogeneity

## Abstract

**Objective:**

This study examined the heterogeneous nature of dual-career stress and its asymmetric associations with on adolescent athletes, aiming to: (1) identify distinct stress profiles based on academic, training, and role-conflict stressors; (2) assess whether stress associations vary across levels of athletic burnout and academic performance; and (3) test whether stress profiles moderate these relationships.

**Methods:**

A two-wave longitudinal study included 843 adolescent male football players in China. Latent Profile Analysis (LPA) categorized participants using three stressor subscales at Time 1. Quantile Regression (QR) at Time 2 (6 months later) analyzed the association between total stress and athletic burnout and academic performance across five quantiles (τ = 0.10–0.90), with stress profile as moderator, controlling for social support, time management, and demographics.

**Results:**

LPA revealed four profiles: Balanced Moderates (37.2%), Academically Overwhelmed (28.1%), Sport-Centric Strained (22.0%), and Dual-Track Distressed (12.7%). QR showed the positive association between stress and burnout increased across quantiles (β = 0.41 at τ = 0.10 to 0.78 at τ = 0.90), with the strongest association observed among already burnt-out athletes most. For academic performance, the negative association between stress and performance was strongest at lower quantiles (β = −0.71 at τ = 0.10) and weaker at higher quantiles (β = −0.29 at τ = 0.90). Stress profiles significantly moderate these relationships: the Dual-Track Distressed profile showed the strongest association with on burnout (β = 0.89), while Academically Overwhelmed and Dual-Track Distressed profiles showed the strongest negative association with on academic performance (β = −0.79 and −0.92, respectively).

**Conclusion:**

Dual-career stress experiences and impacts are highly heterogeneous. Adolescents cluster into meaningful stress profiles, and stress is most strongly associated with negative outcomes among those already at extremes of burnout or poor academic performance. Findings underscore the need for personalized interventions tailored to athletes' specific stress profiles and outcome levels, supporting holistic development in dual-career contexts.

## 1 Introduction

Pursuing a dual career path of sports and education at the same time is a major growth challenge for young athletes. They must move in parallel on the two demanding paths of pursuing elite athletic achievement and achieving good academic results (Stambulova, 2017). Young athletes are increasingly expected to succeed in both fields in the context of policies promoting the integration of sports and education (Condello et al., 2019). However, individuals have limited time, energy, and mental capacity, which in itself can lead to overloading, chronic stress, and potential conflict (Capranica et al., 2022). A large body of research evidence consistently shows that dual occupational stress is a key risk factor for sports burnout, poor academic performance, and eventual withdrawal from sports (Gonçalves et al., 2025; Gustafsson et al., 2017; Cartigny et al., 2021).

Despite this recognition, the mainstream research model suffers from a key oversimplification problem: viewing the stress people feel as something that is exactly the same and has only one dimension, and assuming that it affects all people to a uniform, even degree (Crosswell and Lockwood, 2020). This approach ignores two fundamental issues. First, is there a qualitatively different combination of the multiple occupational pressures borne by young people? Second, does the impact of these stresses vary with the severity of the outcome (e.g., burnout, performance)? That is to say, it will hurt some people greatly and affect others less?

Traditional research methods (variable-centered analysis) often relate a composite stress score to the average level of burnout or achievement, which makes it difficult to answer the above questions (Carroll et al., 2025). They mask the existence of potentially distinct subgroups with distinct patterns of academic stress, sport-specific stress, and role conflict stress (e.g., “academically burdened,” “sport-core,” or “balance-adaptive”) (Adami et al., 2019). Moreover, by focusing only on the average effect, the linear model cannot tell whether the stressor is the “straw that breaks the camel's back” for athletes who are already on the verge of high burnout (at the high end of the distribution). Or whether athletes who are already struggling academically (at the lower end of the distribution) are major disruptors (Carroll et al., 2025).

In order to overcome these limitations, this study proposes an integrated analytical framework, which combines “individual-centered” and “conditional parameterization” research methods (Lv et al., 2022). We first used “latent profile analysis” to identify distinct, data-driven subgroups of youth soccer players based on their performance on multidimensional stress (Deng et al., 2024). This goes beyond merely assessing “how much” the stress is, to categorizing it and looking at “how” the stress is combined (Wang et al., 2023). Subsequently, we integrated these stress patterns into a “quantile regression” model as a key regulatory context (Mutis et al., 2025). This approach allows us to examine how the relationship between cumulative stress and our core outcomes (motor burnout and academic performance) changes as each outcome changes across the conditional distribution (e.g., from low burnout to high burnout). We hypothesized that the effects of stress would be strongest at the high burnout quantile and the low academic performance quantile. Crucially, we believe that the strength of this asymmetric influence is modulated by individual stress patterns, with individuals who are theoretically in the “high-risk” mode showing the most obvious vulnerability.

In addition, recent research on student-athlete migration emphasizes that the experience of dual career stress does not depend solely on objective demands but is shaped by a complex set of push and pull factors, including personal motivation, cultural background, and the availability of support systems (Palumbo et al., 2021; Fuchs et al., 2021). These factors affect how athletes cognitively assess stressors and mobilize coping resources, which are central processes in psychological models of stress and adaptation. Another study showed that perceptions of challenge, effectiveness of support, and performance outcomes differed significantly across countries and genders, implying that the meaning and impact of stress are context-specific (Fuchs et al., 2021). This is consistent with our human-centered research approach. We believe that athletes can distinguish different groups of stress characteristics not only because they face different amounts of stress, but also because they are in different ecological environments, which are shaped by academic requirements, training intensity, role expectations and available resources, thus shaping their different cognitive and emotional responses. By combining latent profile analysis with quantile regression, we go beyond average effects to explore how these characteristic groups moderate the relationship between stress and outcomes at different nodes of burnout and academic performance distributions. Thus, it responds to the call for more detailed and psychological depth of dual career dynamics research (Fusco et al., 2024).

Theoretically, this study integrates the “resource conservation theory” and the life course development model, and holds that different stress patterns reflect different combination States of resource consumption and protection, thus leading individuals to different development trajectories (Singh and Bhuvaneswari, 2023). Methodologically, this study introduces a new synthesis of techniques to simultaneously reveal the heterogeneity of antecedents and the conditionality of consequences in dual career studies (Heo et al., 2024). In practice, the results aim to provide an evidence base for a shift from generic support interventions to personalized strategies tailored to athletes-specific stress patterns, thereby establishing a more precise and effective support system for coaches, parents, and educational institutions (Kimura et al., 2024).

## 2 Methods

### 2.1 Participants and procedure

A longitudinal study design (one semester/training cycle) with two measurements and a six-month interval was used to assess the predictive effect of stressors on subsequent outcomes. Data collection took place from September 2023 to March 2024.

The inclusion criteria were: (1) male soccer players aged 12 to 19; (2) currently receiving systematic academic education and soccer training at the same time; and (3) the participants themselves and their legal guardians had signed a written informed consent. Exclusion criteria were: (1) a significant injury or illness within the last six months that resulted in a suspension from training for more than one month, and (2) a diagnosis of a mental health disorder requiring clinical intervention.

The subjects of the study were young men's football players recruited from 12 football characteristic schools and 8 professional club youth academies in eastern, central and northern China. At the time of the first measurement (T1), a questionnaire was distributed to 913 athletes through the administration of their school or institution. 876 valid T1 questionnaires were retained after eliminating questionnaires due to insufficient time to answer, stereotyped answers, or missing key data. Based on the total number of questionnaires distributed (N = 913), the valid response rate at T1 was 95.9% (876/913). A second measurement (T2) was taken six months later, and these 876 participants were tracked. After successful matching, a longitudinal study sample of 843 people was finally obtained, who had complete data at both time points, accounting for 96.2% of the total sample at the first time point. To assess potential attrition bias, we compared baseline stress levels, age, and years of training between participants who completed both surveys (843) and those who lost after the first time point (33). The results showed no significant differences between the two groups (all *p*-values were greater than 0.05), suggesting that sample attrition was random and unlikely to bias the longitudinal findings.

The mean age of the final sample (N = 843) was 15.84 years (SD = 1.72, range: 12–19 years). The average length of formal physical training was 5.41 years (SD = 2.63). In terms of athlete grade, 37.6% hold the title of national third-class athlete, 28.2% hold the title of national second-class athlete, and 5.1% hold the title of national first-class or higher level (defined as “elite athlete” in this study); the rest are athletes who are systematically trained but have no formal grade certification. We obtained informed consent from all participants and their guardians. The study protocol has been approved by the Ethics Review Committee of Changchun Normal University.

### 2.2 Measures

All scales were in well-validated Chinese and, unless otherwise noted, were on a 5-point Likert scale (1 = strongly disagree, 5 = strongly agree). For all multi-item scales, item scores were averaged to create composite scores, with higher scores indicating greater levels of the measured construct.

**Dual Occupational Stressors (T1):** Using Comprehensive Measures.

**Academic stress** (Chen et al., 2023): The academic burden was measured using a 5-point subscale adapted from the Adolescent Stressors Scale, which has been widely validated as effective (for example, one of the items reads: 'I feel the academic burden is too heavy to bear'). Participants used the Likert 5-point scale to rate each item. This scale has been proven to have good reliability and validity in previous studies targeting Chinese adolescents. In the current sample, its internal consistency reliability is α = 0.87.

**Sport training stress** (Norouzi and Shojaei, 2025): Training-related stress was measured using a condensed version of the Athlete's Training Stressor Scale, which contains four items (for example, “The amount of training makes me physically and mentally exhausted”). This scale has previously been validated in an athlete population. In the current sample, its internal consistency reliability is α = 0.83.

**Role conflict stress** (Sun et al., 2023): Role conflict was measured using the 4-item Student-Athlete Role Conflict Scale (e.g., “I have difficulty meeting the expectations of both coaches and teachers”), which has been validated in a sample of Chinese student-athletes. In the current sample, its internal consistency reliability is α = 0.79.

The criterion scores for the three subscales were used as indicators for subsequent latent profile analysis.

**Exercise burnout (T2)** (Guo et al., 2022): a condensed version of the validated Athlete Burnout Questionnaire, which has been previously validated in the Chinese athlete population, was used for the assessment. The scale contained three dimensions: emotional/physical exhaustion (3 items, α = 0.90), reduced sense of accomplishment (4 items, α = 0.85), and negative evaluation of exercise (3 items, α = 0.81). The total score of burnouts was obtained by calculating the average score of all items, and the higher the score, the more serious the burnout (α = 0.89 for the total scale).

**Academic performance (T2)** (Li et al., 2020): To obtain an objective measure of academic achievement, we collected official school achievement records in core subjects (Chinese, Mathematics, English) for the semester corresponding to the second time point. After receiving permission from the school, the raw scores are converted to in-school standard scores to ensure comparability across schools and grading standards.

**Moderators and covariates (T1):**

**Social Support** (Liu et al., 2023): Perceived social support was measured using the Adolescent Social Support Scale, which assesses support from family, friends, and coaches. The scale consisted of 12 items and was scored on a Likert five-point scale, with higher scores indicating more perceived support. In the current sample, its internal consistency reliability is α = 0.92.

**Time management strategies** (Janeslätt et al., 2018): Time management efficacy was assessed using a validated, 11-item time management efficacy subscale from the Time Management Disposition Inventory. The items were scored by Likert five-point scale, and the higher the score, the stronger the time management ability. In the current sample, its internal consistency reliability is α = 0.79.

Control variables included age, years of training, athlete rank (virtually coded), and a composite index of family socioeconomic status based on parental education and occupation.

### 2.3 Statistical analysis

The analysis was performed using Mplus 8.3 and R 4.2.0 software and followed a two-step integration model.

In order to achieve our objectives of uncovering potential subgroups with different patterns of stress (objective one) and examining whether the effects of stress differ across outcome levels (objective two), we used a two-step, integrated approach. Traditional variable-centered research methods, such as multiple regression, assume that the research population is homogeneous and focus only on the average effect. This may mask the heterogeneity we propose based on resource conservation theory and the life-span development model. Therefore, we first used a person-centered research technique, namely latent profile analysis, to identify different subgroups based on different patterns of stressors. Next, we used the quantile regression method. This approach allows the effects of predictors to be modeled at different points in the distribution of the outcome variable (i.e., different quantiles), thereby testing our hypothesis that the effects of stress are amplified at the extremes of the outcome distribution (e.g., high burnout or low academic performance).

Step 1: Potential Profile Analysis (LPA). The first step of the analysis was designed to identify heterogeneous subgroups based on the pattern of stressors at T1. Using the Mplus software, a series of latent profile models ranging from 1 to 6 profiles were estimated, with three standardized stressor subscales (academic, training, role conflict) as continuous indicators. Model selection is based on a comprehensive assessment of statistical fit indices and theoretical interpretability. Specifically, we compare the Akaike Information Criterion (AIC), the Bayesian Information Criterion (BIC), and the sample-adjusted BIC (aBIC), with lower values indicating better fit. We also examined the entropy value (greater than 0.80 indicates clear classification), the Lo-Mendell-Rubin adjusted likelihood ratio test (LMR-LRT), and the bootstrap likelihood ratio test (BLRT). The p-value of the k-class model significantly indicates that it fits better than k-1 class model (Nylund-Gibson et al., 2023). LPA does not require the same parametric assumptions (e.g., normality, homogeneity of variance) as traditional inferential tests; model selection was based on multiple fit indices and interpretability, as recommended in the methodological literature. The final model was selected to balance best fit, parsimony, class proportions (> 5%), and the uniqueness and significance of each profile feature. For subsequent analysis, the most likely profile attribution for each participant was saved and derived.

Step 2: Quantile regression with the profile as the moderator. The second step examined the heterogeneous effect of pressure on different outcome distributions, with the LPA-derived profiles as moderating variables. Quantile regression was chosen because it makes no distributional assumptions about the error term and is robust to heteroscedasticity and outliers, which is particularly important when examining effects across the full outcome distribution. Quantile regression models were constructed for motor burnout (total score) and academic performance (Z-score), respectively, for T2, using the Quantreg package in R language. The core independent variable was the total stress score of T1 (the mean of the three subscales). Categorized profile variables were effect coded, converted to dummy variables, and the profile with the largest number of people was used as the reference group. The base model incorporated the interaction of total stress with the profile dummy variable (formally tested by Wald test interaction terms) while controlling for social support, time management strategies, age, years of training, athlete rank, and socioeconomic status. The model was estimated at five key quantiles of the outcome distribution: τ = 0.10, 0.25, 0.50, 0.75, and 0.90 (Lee et al., 2021). To obtain robust standard errors and confidence intervals that do not rely on parametric assumptions, we used the bootstrap method with 1,000 replications. Results are presented through tables of coefficients at key quantiles, and graphs depicting the trajectory of the pressure coefficient and its 95% confidence interval for each outcome over a range of quantiles (from τ = 0.05 to 0.95). For significant interaction effects, a Wald test was performed to compare the differences in pressure coefficients between different profiles. A simple slope plot showing the relationship of different profile attributions downforce to outcome was generated for a specific quantile (e.g., τ = 0.75 for burnout).

The sample size of our study was 843, which was statistically powerful enough to perform both latent profile analysis and quantile regression analysis. This sample size is well above the standard typically recommended for identifying meaningful latent profiles and provides stable estimates across multiple quantiles and with multiple covariates. Regarding the issue of multiple comparisons, since our focus is on the variation pattern of coefficients in different quantiles and different characteristic groups, rather than isolated significance tests, and all reported results are consistent with a coherent theoretical model, we do not use formal multiple comparison correction methods such as Bonferroni correction, as this would be too conservative. Increase the likelihood of making Type II errors. Instead, we focus on effect sizes, confidence intervals, and overall outcome patterns across the entire outcome distribution.

Step 3: Robustness test. Several additional analyses are planned to assess the robustness of the study results. First, the stability of the LPA solution was examined by re-running the analysis using the factor scores of the stressor subscale as an indicator. Secondly, the sensitivity of quantile regression results is tested by estimating the model on different combinations of quantiles. Third, we performed a Wald test to examine the statistical significance of the difference in slope between different quantiles. Finally, a critical analysis was repeated using a subjective measure of academic satisfaction in lieu of an objective Z-score for academic performance to ensure that the findings were not an artifact of the way a particular outcome was operationalized.

## 3 Results

### 3.1 Descriptive statistics and stress profile identification

The preliminary analysis identified the basic relationships between the study variables (see Table 1). Higher total stress at T1 was significantly associated with greater motor burnout (*R* = 0.54, *p* < 0.001) and worse academic performance (*R* = −0.38, *p* < 0.001) at T2. Social support and time management strategies are negatively correlated with stress and burnout, and positively correlated with academic performance, indicating that they are key protective factors.

**Table 1:** Sample characteristics and stress profile comparisons.

**Variable**	**Full sample (*N* = 843)**	**Balanced moderates (*n* = 313, 37.1%)**	**Academically overwhelmed (*n* = 237, 28.1%)**	**Sport-centric strained (*n* = 185, 21.9%)**	**Dual-track distressed (*n* = 108, 12.8%)**	**Statistical test *p*-value**
**Demographics**						
Age (years)	15.84 ± 1.72	15.91 ± 1.69	15.87 ± 1.71	15.78 ± 1.75	15.62 ± 1.79	0.138
Family SES (z-score)	0.00 ± 0.85	0.08 ± 0.81	−0.05 ± 0.87	0.02 ± 0.84	−0.21 ± 0.92	< 0.001
**Sport background**						
Training years	5.41 ± 2.63	5.52 ± 2.58	5.38 ± 2.61	5.47 ± 2.69	5.02 ± 2.74	0.093
Elite athletes (%)	43 (5.1%)	18 (5.8%)	11 (4.6%)	9 (4.9%)	5 (4.6%)	0.942
**T1 Stressors**						
Academic stress	3.12 ± 0.88	2.51 ± 0.62	4.18 ± 0.43	2.98 ± 0.71	4.52 ± 0.38	< 0.001
Training stress	2.88 ± 0.81	2.29 ± 0.58	2.65 ± 0.63	3.87 ± 0.52	3.94 ± 0.47	< 0.001
Role conflict	2.73 ± 0.92	1.98 ± 0.61	2.85 ± 0.74	3.12 ± 0.82	4.32 ± 0.49	< 0.001
**T1 protective factors**						
Social support	3.65 ± 0.89	3.92 ± 0.81	3.58 ± 0.87	3.62 ± 0.90	3.12 ± 0.94	< 0.001
Time management	3.22 ± 0.77	3.41 ± 0.72	3.18 ± 0.75	3.15 ± 0.79	2.84 ± 0.82	< 0.001
**T2 outcomes**						
Athletic burnout	2.47 ± 0.82	2.08 ± 0.71	2.42 ± 0.78	2.68 ± 0.85	3.24 ± 0.88	< 0.001
Academic performance (z)	0.02 ± 0.98	0.18 ± 0.91	−0.15 ± 0.95	0.08 ± 1.02	−0.41 ± 1.04	< 0.001

**Figure 1:**
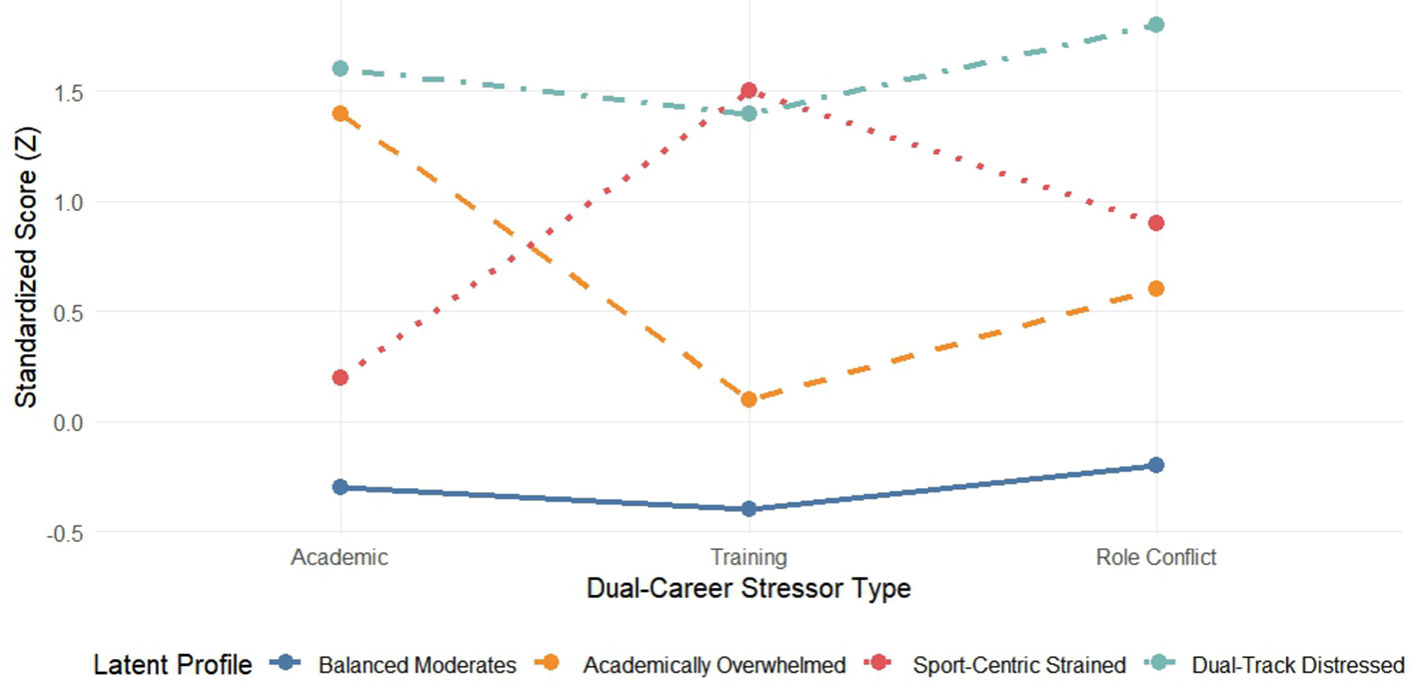
Characteristics of four potential pressure profiles. This figure shows the standardized scoring patterns of four types of young football players identified by latent profile analysis on three stressors: academic, training and role conflict. Each profile represents a unique type of pressure combination: “balance adaptation type” is low in all kinds of pressure; “academic burden type” is particularly prominent in academic pressure; “sports core pressure type” is dominated by training pressure; “dual-track damage type” is at a high level in all pressure sources.

Latent profile analysis (LPA) for the three T1stressor indicators resulted in a robust 4-profile model with excellent classification accuracy (entropy = 0.88) and superior fit index (see Table 2) as the optimal solution. As shown in Figure 1, these four profiles have a clear degree of differentiation: the “balanced adaptation” group (37.2%), which has relatively low pressure in all areas; the “academic burden” group (28.1%), which is characterized by extremely high academic pressure; The “sports core stress” group (22.0%), which was dominated by training stress (accompanied by high role conflict), and the high-risk “dual-track” group (12.7%), which showed severe elevation in all stressors. *Post-hoc* tests (ANOVA with Tukey HSD correction) confirmed significant differences across profiles in all pressure dimensions (all *p* < 0.001, see Table 1). These profiles also differed significantly in protective resources and outcomes, with the “dual-track” group reporting the lowest social support and the worst outcomes.

**Table 2:** Model fit indices for latent profile analysis (LPA).

**Number of Profiles (k)**	**Log-Likelihood**	**AIC**	**BIC**	**aBIC**	**Entropy**	**LMR-LRT *p*-value**	**BLRT *p*-value**
1	−2,472.51	4,955.02	4,974.92	4,962.67	–	–	–
2	−2,358.73	4,735.46	4,769.71	4,748.43	0.83	< 0.001	< 0.001
3	−2,310.24	4,644.48	4,693.08	4,662.77	0.86	< 0.001	< 0.001
4	−2,285.09	4,598.18	4,661.13	4,621.79	0.88	0.002	< 0.001
5	−2,276.58	4,585.16	4,662.46	4,614.08	0.87	0.126	0.064
6	−2,269.42	4,574.84	4,666.49	4,609.08	0.88	0.085	0.053

AIC, Akaike Information Criterion; BIC, Bayesian Information Criterion; aBIC, sample-size-adjusted BIC; LMR-LRT, Lo-Mendell-Rubin adjusted Likelihood Ratio Test; BLRT, Bootstrap Likelihood Ratio Test. Bold values indicate the selected 4-profile model.

Data presented as M ± SD or n (%). Bold values indicate significantly different from other profiles based on post-hoc tests (Tukey HSD, p < 0.05). Statistical tests: ANOVA for continuous variables, χ^2^ for categorical.

### 3.2 Heterogeneous effects of stress: quantile regression results

The results of the quantile regression analysis (summarized in Table 3) showed significant heterogeneity in the conditional distribution of the effects of total stress on each outcome.

**Table 3:** Quantile regression results: heterogeneous effects of stress.

**Outcome/Quantile**	**τ = 0.10**	**τ = 0.25**	**τ = 0.50**	**τ = 0.75**	**τ = 0.90**	**OLS**
**Athletic burnout**						
Total stress	0.41^***^ (0.06)	0.48^***^ (0.05)	0.57^***^ (0.04)	0.68^***^ (0.05)	0.78^***^ (0.07)	0.59^***^ (0.03)
Social support	−0.12^**^(0.04)	−0.15^***^(0.03)	−0.18^***^(0.03)	−0.21^***^ (0.03)	−0.24^***^ (0.05)	−0.19^***^ (0.02)
Time management	−0.08^*^(0.04)	−0.11^***^(0.03)	−0.14^***^(0.03)	−0.17^***^(0.03)	−0.20^***^ (0.05)	−0.15^***^ (0.02)
Pseudo-*R*^2^	0.29	0.32	0.36	0.39	0.42	0.37
**Academic Performance**						
Total stress	−0.71^***^(0.08)	−0.62^***^(0.06)	−0.48^***^(0.05)	−0.38^***^(0.06)	−0.29^**^(0.09)	−0.51^***^(0.04)
Social support	0.09^*^(0.05)	0.11^**^(0.04)	0.13^***^(0.03)	0.15^***^(0.04)	0.17^**^(0.06)	0.12^***^(0.03)
Time management	0.21^***^(0.05)	0.24^***^(0.04)	0.27^***^(0.03)	0.30^***^(0.04)	0.33^***^(0.06)	0.26^***^(0.03)
Pseudo-R^2^	0.34	0.32	0.29	0.25	0.22	0.30

Standard errors in parentheses. All models control for age, training years, athletic level, and SES. Pseudo-R^2^ calculated using Koenker-Machado method. ^*^p < 0.05, ^**^p < 0.01, ^***^p < 0.001.

For exercise burnout, the positive coefficient of stress showed a significant monotonic increase from the lower quantile to the higher quantile. This suggests that the negative effects of stress are significantly amplified for athletes who already experience higher levels of burnout (β _ {τ = 0.10} = 0.41 vs. β _ {τ = 0.90} = 0.78), This pattern was confirmed by the Wald test for slope heterogeneity [χ^2^ (4) = 28.45, *p* < 0.001].

**Figure 2:**
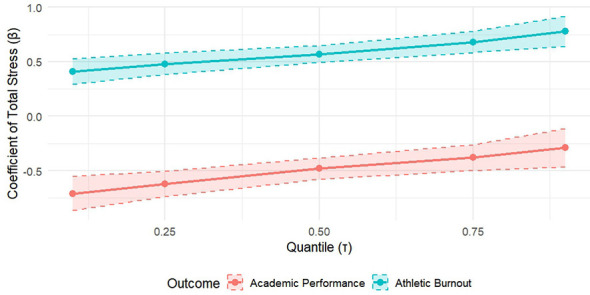
Effect of total pressure on the results at different quantiles. This figure depicts the trajectory of the total stress coefficient (β) and its 95% confidence interval on the conditional distribution of exercise burnout versus academic performance (τ = 0.10 to 0.90) after controlling for covariates. The solid line represents the trend of the pressure coefficient as a function of the quantile, and the shaded area indicates the confidence interval. For exercise burnout (orange), the stress effect was stronger at the high quantile (high burnout level); for academic performance (blue), the stress effect was stronger at the low quantile (low academic level). The dotted line represents the estimate of the traditional OLS regression (single mean) for comparison.

In contrast, for academic performance, the negative effect of stress was strongest at the low end of the performance distribution and waned toward the high end. This suggests that stress is particularly damaging to the academic outcomes of athletes who are already academically vulnerable (β _ {τ = 0.10} = −0.71 vs. β _ {τ = 0.90} = −0.29). The full trajectory of these differential effects is shown in Figure 2, emphasizing the non-uniformity of the pressure effect, which is obscured by the traditional mean-based least squares (OLS) estimation.

### 3.3 Regulation of potential pressure profile

The relationship between pressure and outcome was significantly moderated by the attribution of the underlying profile. Stratified analysis at the key quantile (see Table 4) showed that for exercise burnout at τ = 0.75 (higher burnout level), the effect of stress followed a clear risk gradient: it was weakest for the “balanced adaptation” group (β = 0.52), moderate for the “academically stressed” group (β = 0.61), It was stronger in the “exercise core pressure type” group (β = 0.74), and the strongest in the “dual-track loss type” group (β = 0.89). Wald tests for interaction terms confirmed that the stress effect was significantly stronger for all high-risk profiles than for the balanced group (all *p* < 0.01).

**Table 4:** Moderation by stress profile: stratified quantile regression.

**Stress profile**	**Athletic burnout at τ = 0.75**	**Academic performance at τ = 0.25**	**Significance tests**
Balanced moderates (*n* = 313)	0.52^***^[0.44, 0.60]	−0.41^***^[−0.53, −0.29]	Reference
Academically overwhelmed (*n* = 237)	0.61^***^[0.51, 0.71]	−0.79^*^[−0.95, −0.63]^**^	vs. Balanced: χ^2^ = 25.7^***^
Sport-centric strained (*n* = 185)	0.74^*^[0.64, 0.84]^**^	−0.55^***^[−0.71, −0.39]	vs. Balanced: χ^2^ = 22.2^***^
Dual-track distressed (*n* = 108)	0.89^*^[0.76, 1.02]^**^	−0.92^*^[−1.14, −0.70]^**^	vs. Balanced: χ^2^ = 45.7^***^
**Profile comparisons**			
High-risk vs. Balanced	χ^2^(3) = 68.3^***^η^2^ = 0.075	χ^2^(3) = 48.9^***^ η^2^ = 0.055	
Moderation effect size	Δ*R*^2^ = 0.048, *f*^2^ = 0.051	Δ*R*^2^ = 0.036, *f*^2^ = 0.038	

Coefficients for total stress with 95% confidence intervals in brackets. High-risk profiles (Academically Overwhelmed, Sport-Centric Strained, Dual-Track Distressed) show stronger effects. Moderation effect size indicates the variance explained by profile × stress interaction. ^*^p < 0.05, ^**^p < 0.01, ^***^p < 0.001.

**Figure 3:**
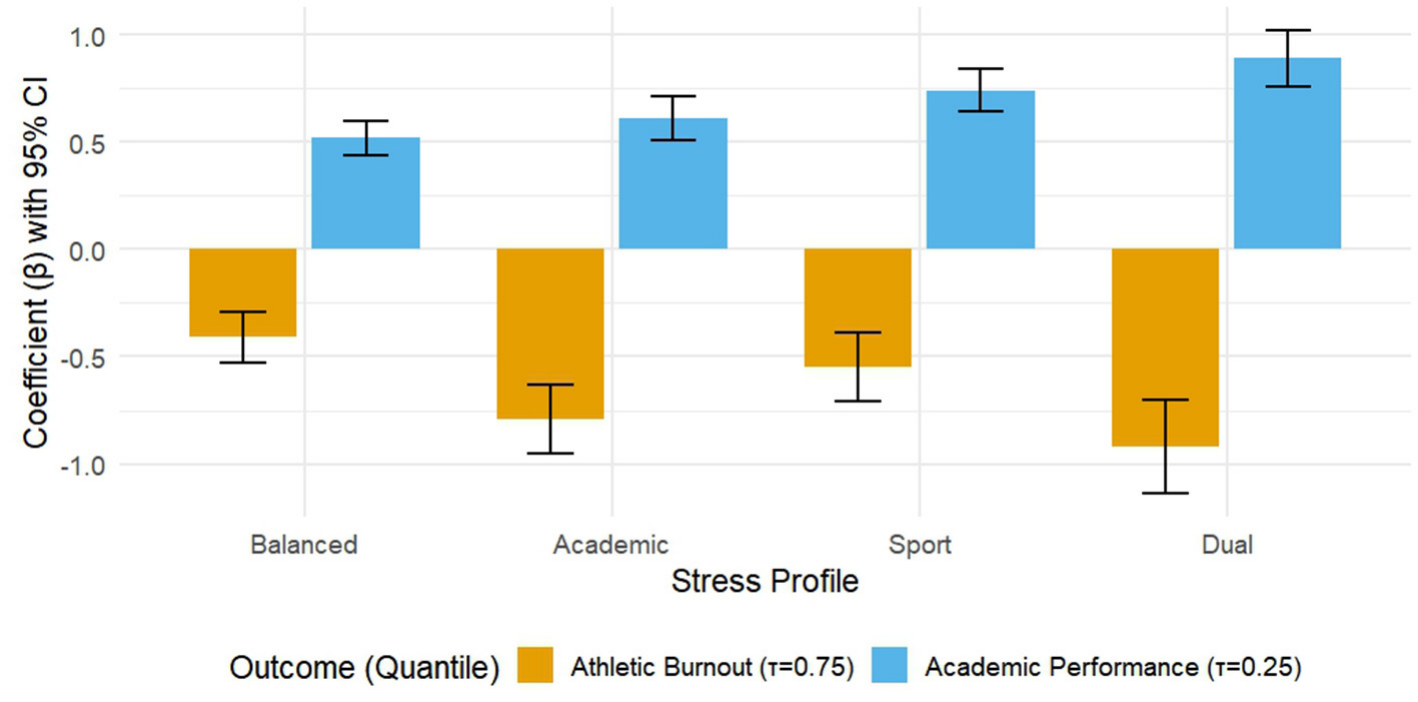
Moderating effects of different pressure profiles at key quantile points. This figure shows the difference in the effect values (beta coefficients) of the total pressure on the results for different pressure profiles at a particular quantile. The left half (τ = 0.75) shows the effect of stress on motor burnout, and the right half (τ = 0.25) shows the effect of stress on academic performance. The column height represents the β coefficient, and the error bar is the 95% confidence interval. The results showed that the “dual-track” profile had the highest risk of exacerbating burnout, while the “academic burden” and “dual-track” profiles had the greatest impact on academic performance.

For academic performance at τ = 0.25 (lower academic level), a different regulation pattern emerges. Here, the most serious negative effects of stress are concentrated in those profiles characterized by high academic stress, namely, the “academically stressed” group (β = −0.79) and the “dual-track” group (β = −0.92). Their coefficients were significantly greater than those for the “moving core pressure” and “balanced adaptation” profiles (Wald test for interaction: χ^2^ values from 22.2 to 45.7, all *p* < 0.001). These conditional relationships, shown in Figure 3, suggest that the specific combination of stressors experienced by athletes fundamentally shapes how general stress levels translate into negative outcomes.

### 3.4 Robustness test

A series of complementary analyses confirmed the robustness of the main findings (summarized in Table 5). The LPA solution for the 4-profile remains stable when alternative estimation methods are used. The pattern of quantile regression coefficients remained consistent when using different sets of quantiles or controlling for additional covariates (e.g., parental support). Subgroup analysis by age and exercise level indicated that the basic pattern of heterogeneous effects and profile modulation persisted despite changes in effect size. Sensitivity analyses using alternative stress measures (e.g., composite indices, PSS-10 scores) yielded highly correlated results (*R* > 0.89), supporting the validity of the main findings.

**Table 5:** Robustness checks and sensitivity analyses.

**Test type**	**Model specification**	**Key finding**	**Consistency**
**Measurement robustness**			
Composite stress index	Weighted factor scores	Similar coefficient patterns	High (*r* = 0.94)
Alternative stress scale	PSS-10 total score	Comparable effect sizes	High (*r* = 0.89)
**Model specification**			
Extended controls	Parental support, school climate	Main effects robust (Δβ < 5%)	High
Nonparametric QR	Local polynomial smoothing	Similar coefficient trajectories	High
**Subgroup analyses**			
By age group	Younger (12–15) vs. older (16–19)	Stronger effects in younger group	Moderate
By athletic level	Elite vs. non-elite	Different patterns by outcome	Moderate
**Mechanism exploration**			
Mediation analysis	Stress → Social support → Outcomes	Partial mediation (15.2% of total effect)	–
Interaction test	Stress × Time management	Buffering effect significant	–

Consistency ratings: High = correlation >0.85 with main results; Moderate = correlation 0.70–0.85. All sensitivity tests confirm the robustness of main findings.

## 4 Discussion

This study used an approach that integrates individual-centered and conditional parameterization to deepen the understanding of dual career stress in youth soccer players. By combining latent profile analysis (LPA) and quantile regression (QR), we remedy two key blind spots of previous studies: the assumption that stressors are homogeneous, and the limitation of focusing only on the average effect. The results provide strong evidence that the patterns of adolescents experiencing dual occupational stressors are qualitatively distinct, and that the effects of these stressors are uneven, producing significant differences depending on the individual's existing level of burnout, academic performance, and their specific combination of stressors.

### 4.1 Main findings and theoretical implications

Our primary contribution is the empirical identification of four different stress profiles — balance adaptation, academic overload, athletic core stress, and dual track impairment (Stattin et al., 2025). This finding has echoes and extensions of LPA research in the context of dual occupations in Europe. For example, similar studies have identified “well-adjusted” and “multiple-stressed” groups (Cartigny et al., 2021; Gonçalves et al., 2025). However, the “academic burden” profile found in the sample of Chinese young football players in this study is particularly prominent, which may reflect that academic requirements constitute a unique and heavy pressure dimension in the collectivist cultural context that emphasizes academic achievement.

In the Chinese sample, the emergence of the feature of “excessive academic burden” may also reflect the cultural particularity of dual career expectations. A previous study reported that there are significant differences in the migration experience of European student athletes between countries, which are reflected in economic support, performance decline and willingness to migrate (Fuchs et al., 2021). These findings underscore the idea that the experience of dual career stress is not constant across cultures. Instead, it will be filtered and influenced by social values, educational systems and sports policies. In China, the high social emphasis on academic achievement, coupled with the policy orientation of promoting the integration of sports and education, may magnify the academic pressure faced by even elite athletes. This cultural perspective adds complexity to the psychological level, which means that the formation of stress characteristics depends not only on objective requirements, but also on the internalized expectations and social comparisons formed by athletes according to their own cultural environment. Future cross-cultural research should examine whether similar identity groups emerge in other national contexts and how cultural values mediate the relationship between stress and outcomes.

This classification goes beyond a one-dimensional perspective that focuses only on the “amount” of stress and moves to delineate specific “combinations” of stressors (Zhou et al., 2025). This finding strongly supports the idea, derived from the resource conservation theory (COR), that individuals experience resource depletion in a patterned manner (Lin et al., 2022). For example, the academically burdened and athletically stressed profiles represent specialized channels of resource depletion whose demands primarily consume resources in one area of life (Chen, 2024). It is worth noting that the core stress profile of sports also shows high role conflict, which indicates that high-intensity sports input itself will produce tension with academic roles (Mateu et al., 2020). This finding can be further explained from the perspective of self-determination theory: when athletes over-identify with and devote themselves to the role of athletes, their opportunities to meet the basic psychological needs of autonomy (such as the sense of choice of academic path), ability (such as the sense of achievement in academic achievement) and belonging (such as integration into the peer group of non-athletes) may be squeezed. It intensifies the internal conflict and tension between the characters, not just the external competition in time.

The identification results of these four characteristics are consistent with the multi-level ecological perspective theory proposed by previous studies (Fusco et al., 2024). The theory divides dual career support into three levels: micro (individual level), meso (interpersonal/organizational level) and macro (policy/cultural level). In our study, the group of “moderate balance” probably reflects good coordination among these three levels, which is manifested in having sufficient personal resources (micro level), being supported by coaches and teachers (meso level), and being able to consider the dual needs of the school or sports environment (macro level). In contrast, the “two-track frustration” group may represent problems at all levels, including resource exhaustion at the individual level, role conflict in interpersonal relationships, and lack of flexibility at the organizational level. Another previous study provided support for this interpretation, finding that migrant student-athletes' perceptions of declining academic and athletic performance were largely influenced by meso-and macro-level factors such as tutoring support, organizational help, and financial assistance (Fuchs et al., 2021). Therefore, these characteristic groups we identified should not only be regarded as statistical classifications, but also reflect the deep psychological processes generated by the interaction between individual characteristics and environmental support, such as perceived control, self-efficacy and coping efficacy. Future studies should directly measure these psychological mechanisms in order to uncover the “black box” connecting stress characteristics with final outcomes.

In contrast, the dual-track lossy profile represents a long-term and pervasive state of resource depletion across domains, which is likely to trigger a spiral of depletion leading to the worst outcomes (Keane et al., 2020). While the balanced adaptive profile reports the highest levels of protective resources (social support, time management) and may benefit from a more efficient resource input and gain spiral (Neveu and Bégout, 2025).

The second important contribution lies in revealing the nonlinear and asymmetric associations with pressure. Quantile regression results show that stress is most strongly positively associated with athletes who are already in the high burnout quantile, while it is most strongly negatively associated with athletes who are in the low academic performance quantile (Sherwood et al., 2016). This pattern challenges linear models and fits with the concept of “tipping point” or “vulnerability amplification” (Beauchaine and Hinshaw, 2020). It suggests that additional stress has a disproportionately stronger association once an athlete's resource reserves are severely depleted (high burnout) or developmental pathways are severely compromised (low academic performance) (Cai et al., 2025). This finding highlights the importance of looking at the entire distribution of outcomes, as the key dynamics tend to be concentrated in the extreme parts of the distribution.

Most importantly, this study demonstrates that these two types of heterogeneity — antecedent heterogeneity (profile) vs. consequence heterogeneity (quantile effects) are interrelated. The pressure profile significantly modulates the relationship between pressure and outcome. The amplification effect of stress on high burnout is most severe in those profiles characterized by high exercise-related or global stress (exercise-core stress type, dual-track type). The erosive effect of stress on low academic performance is concentrated in those profiles characterized by high academic stress (academic burden type, dual-track damage type) (Quinaud et al., 2021). This interaction provides a more nuanced, individual and contextual explanation: the type of combination of stressors experienced by individuals guides and is associated with the effects of overall stress levels on specific vulnerabilities (Waldén et al., 2023).

### 4.2 The practical significance of supporting athletes

The integrated LPA-QR framework goes beyond previous generalized approaches to provide a precise and actionable blueprint for personalized support in dual career contexts (Fegert et al., 2020). Unlike previous studies that typically advocate uniform stress management strategies based on overall stress levels, our findings call for a diagnostic, profile-specific approach (Rice et al., 2019). For practitioners (coaches, consultants), the first key step is to move from assessing “how much” an athlete reports stress to diagnosing “how it is structured”-for example, Distinguish between the “academically stressed” who need academic coaching and deadline management and the “sports core stress” who need training load monitoring and autonomous supportive coaching (Ojala et al., 2023). This represents a significant shift from general resilience training to precision interventions (Ekelund et al., 2022). Therefore, the support system must be reconceptualized as a hierarchical model: providing universal health development promotion for the balanced adaptive type, providing targeted and domain-specific skill development for the academically burdened and athletically stressed type,; And immediate and intensive case management is needed for high-risk “dual-track” groups, whose multiple resource losses require systematic intervention (Thompson et al., 2022). In addition, quantile regression results add a key temporal dimension that was missing from previous mean-based models (Purcell et al., 2022). They suggest that the urgency and focus of the intervention must be calibrated to the athletes' position on the continuum of outcomes: prioritizing crisis-level stress relief for those near the high burnout threshold and a solid academic support architecture for those in the low performance quantile (Gouttebarge et al., 2018). Ultimately, these insights call for policy innovation that urges sport and education institutions to move beyond the one-size-fits-all model of care and instead create flexible policy frameworks that identify and institutionally embrace these empirically confirmed risk profiles, thereby fostering an ecosystem that can underpin both athletic talent and academic development (Baker et al., 2017).

### 4.3 Limitations and future research directions

There are some limitations in this study that need to be explained. One, although this is a longitudinal study, the design of only two time points does not conclusively establish causality. The main limitation is the two-wave longitudinal design, which offers advantages over cross-sectional data but does not allow for causal inference. Some unmeasured third-party variables, such as personality traits and genetic factors, may explain the observed relationships. Future studies should employ more intensive longitudinal methods, such as empirical sampling, or experimental designs to examine causal pathways. Second, our sample contains only male soccer players, which limits the generalization of the results to female athletes, other sports, and different cultural backgrounds. The specific stress characteristics identified may be influenced by the Chinese education and sports system, which emphasizes academic achievement. Future research should examine the prevalence of these stress characteristics across gender, sport, and cultural contexts. Third, reliance on self-reported measures, while common, may introduce bias. Despite our use of validated scales, self-report measures suffer from recall bias and common methodological variation. Future research should incorporate multivariate assessment methods, such as interviews, coach or parent reports, and physiological indicators of stress. The inclusion of objective measures, such as cortisol levels, actual training load, and official performance, will enhance the persuasiveness of future studies.

The current findings open several promising research directions. First, a potential transition analysis should be used to explore the stability and transition of the pressure profile over time. Do athletes switch between different profiles? What triggers these shifts? Second, research should explore specific mechanisms (e.g., coping styles, motivational climate, coach-athlete relationships) that explain why some profiles are more vulnerable than others. Third, intervention studies can now be designed to examine whether support programs tailored to athletes-specific stress profiles are more effective than standard programs. Fourth, the definition of “elite athletes” (5.1% with a national level or higher) may limit the generalization of the findings to other levels of sport. Future studies should incorporate more diverse performance hierarchies. Fifth, despite our analysis of attrition, the possibility of unmeasured confounders remains.

## 5 Conclusion

This study demonstrates that adolescent athletes' experience of dual career stress and its outcomes are fundamentally heterogeneous. By integrating an individual-centered and distribution-sensitive approach, we find that adolescents cluster according to meaningful stressor patterns, and that stress is most strongly associated with those individuals who are already at the negative extremes of burnout or academic performance. Crucially, the stressor profile of the individual is related to this relationship, revealing a complex interplay between the structure of needs and developmental outcomes.

Our research shows that the impact of stress depends on which characteristic group an individual belongs to and at which outcome level. This finding contributes to a growing body of scholarly literature that calls for context-sensitive, multilevel approaches to understanding psychological adaptation processes under stress. Combining the two research methods of “people-centered” and “distribution-sensitive” provides a model for future research, which helps to reveal the multiple paths of occupational and academic stress affecting individual wellbeing and performance. From a psychological perspective, our findings highlight a key point, namely that the “same” level of stress can lead to dramatically different outcomes. This depends on the resource reserve that athletes already have (which is reflected in their position in the distribution of results, that is, quantiles) and the specific combination of pressures they face (which is reflected in their type of pressure characteristics). This highlights the importance of going beyond traditional models that focus on variables and only average effects. We should have a deeper understanding of how individual cognitive assessment, coping efficacy and perceived control interact with the support provided by the environment, and ultimately lead to well-adapted or maladaptive development trajectories.

These insights provide a solid empirical foundation for developing targeted, effective, and equitable support systems that recognize and respond to the diverse realities of current adolescent dual career experiences. Going forward, the adoption of this nuanced, personalized approach is critical to fostering superior athletic performance and overall development among young athletes.

## Data Availability

The raw data supporting the conclusions of this article will be made available by the authors, without undue reservation.
